# Discriminative topological features reveal biological network mechanisms

**DOI:** 10.1186/1471-2105-5-181

**Published:** 2004-11-22

**Authors:** Manuel Middendorf, Etay Ziv, Carter Adams, Jen Hom, Robin Koytcheff, Chaya Levovitz, Gregory Woods, Linda Chen, Chris Wiggins

**Affiliations:** 1 Department of Physics, Columbia University, New York, USA; 2 College of Physicians and Surgeons, Columbia University, New York, USA; 3 Columbia College, Columbia University, New York, USA; 4 Fu Foundation School of Engineering and Applied Sciences, Columbia University, New York, USA; 5 Barnard College, Columbia University, New York, USA; 6 Department of Mathematics, Columbia University, New York, USA; 7 Department of Applied Physics and Applied Mathematics, Columbia University, New York, USA; 8 Center for Computational Biology and Bioinformatics, Columbia University, New York, USA

## Abstract

**Background:**

Recent genomic and bioinformatic advances have motivated the development of numerous network models intending to describe graphs of biological, technological, and sociological origin. In most cases the success of a model has been evaluated by how well it reproduces a few key features of the real-world data, such as degree distributions, mean geodesic lengths, and clustering coefficients. Often pairs of models can reproduce these features with indistinguishable fidelity despite being generated by vastly different mechanisms. In such cases, these few target features are insufficient to distinguish which of the different models best describes real world networks of interest; moreover, it is not clear a priori that *any *of the presently-existing algorithms for network generation offers a predictive description of the networks inspiring them.

**Results:**

We present a method to assess systematically which of a set of proposed network generation algorithms gives the most accurate description of a given biological network. To derive discriminative classifiers, we construct a mapping from the set of all graphs to a high-dimensional (in principle infinite-dimensional) "word space". This map defines an input space for classification schemes which allow us to state unambiguously which models are most descriptive of a given network of interest. Our training sets include networks generated from 17 models either drawn from the literature or introduced in this work. We show that different duplication-mutation schemes best describe the *E. coli *genetic network, the *S. cerevisiae *protein interaction network, and the *C. elegans *neuronal network, out of a set of network models including a linear preferential attachment model and a small-world model.

**Conclusions:**

Our method is a first step towards systematizing network models and assessing their predictability, and we anticipate its usefulness for a number of communities.

## 1 Background

The post-genomic revolution has ushered in an ensemble of novel crises and opportunities in rethinking molecular biology. The two principal directions in genomics, sequencing and transcriptome studies, have brought to light a number of new questions and forced the development of numerous computational and mathematical tools for their resolution. The sequencing of whole organisms, including *homo sapiens*, has shown that in fact there are roughly the same number of genes, for example, in mice and men. Moreover, much of the coding regions of the chromosomes (the subsequences which are directly translated into proteins) are highly homologous. The complexity comes then, not from a larger number of parts, or more complex parts, but rather through the complexity of their interactions and interconnections.

Coincident with this biological revolution – the massive and unprecedented volume of biological data – has blossomed a technological revolution with the popularization and resulting exponential growth of the computing networks. Researchers studying the topology of the Internet [1] and the World Wide Web [2] attempted to summarize these topologies via statistical quantities, primarily the distribution *P*(*k*) over nodes of given connectivity or degree *k*, which was found to be completely unlike that of a "random" or Erdös-Rényi graph. Instead, the distribution obeyed a power-law *P*(*k*)~*k*^-*γ*^. As a consequence many mathematicians concentrated on (i) measuring the degree distributions of many technological, sociological, and biological graphs (which generically, it turned out, obeyed such power-law distributions) and (ii) proposing various models of randomly-generated graph topologies which could reproduce these degree distributions (*cf*. [3] for a thorough review). The success of these latter efforts reveals a conundrum for mathematical modeling: a metric which is universal (rather than discriminative) cannot be used for choosing the model which best describes a network of interest. The question posed is one of *classification*, meaning the construction of an algorithm, based on training data from multiple classes, which can place data of interest within one of the classes with small test loss.

Systematic enumeration of substructures has so far been used to find statistically significant subgraphs or "motifs" [4-8] by comparing the network of interest to an assumed null model. Recently, the idea of clustering real networks into groups based on similarity in their "significance profiles" has been proposed [9]. We here use and extend these ideas to compare a given network of interest to a set of proposed network models. Rather than unsupervised clustering of real networks, we perform supervised classification of network models. In this paper, we present a natural mapping from a graph to an infinite-dimensional vector space using simple operations on the adjacency matrix. The coordinates (called "words", see Methods) reflect the number of various substructures in the network (see Figures 3 and 6). We then use support vector machines (SVMs) to build classifiers that are able to discriminate different network models. The performance of these classifiers is measured using the empirical test-loss on a hold-out set, thus estimating the probability of misclassifying an unseen test network. We selected 17 different mechanisms proposed in the literature to model various properties of naturally occurring networks. Among them are various biologically-inspired graph-generating algorithms which were put forward to model genetic or protein interaction networks. We are then able to classify naturally occurring networks into one of the proposed classes. We here classify data sets for the *E. coli *genetic network, the *C. elegans *neuronal network and the yeast *S. cerevisiae *protein interaction network. To interpret and understand our results further we define a measure of robustness to estimate the confidence of the resulting classification. Moreover, we calculate *p*-values using Gaussian kernel density estimation to find substructures that are characteristic of the network model or the real network of interest. We anticipate that this new approach will provide general tools of network analysis useful to a number of communities.

## Results and Discussion

We apply our method to three different real data sets: the *E. coli *genetic network [10] (directed), the *S. cerevisiae *protein interaction network [11] (undirected), and the *C. elegans *neuronal network [12] (directed).

Each node in *E. coli*'s genetic network represents an operon coding for a putative transcriptional factor. An edge exists from operon *i *to operon *j *if operon *i *directly regulates *j *by binding to its operator site. This gives a sparse adjacency matrix with a total of 423 nodes and 519 edges.

The *S. cerevisiae *protein interaction network has 2114 nodes and 2203 undirected edges. Its sparseness is therefore comparable to that of *E. coli*'s genetic network.

The *C. elegans *data set represents the organism's fully mapped neuronal network. Here, each node is a neuron and each edge between two nodes represents a functional, directed connection between two neurons. The network consists of 306 neurons and 2359 edges, and is therefore about 7 times more dense than the other two networks. We create training data for undirected or directed models according to the real data set. All parameters other than the numbers of nodes and edges are drawn from a uniform distribution over their range. We sample 1000 examples per model for each real data set, train a pairwise multi-class SVM on 4/5 of the sampled data and test on the 1/5 hold-out set. We determine a prediction by counting votes for the different classes. Table 1 summarizes the main results. All three classifiers show very low test loss and two of them a very high robustness (see Subsection Robustness under Methods). The average number of support vectors is relatively small. Indeed, some pairwise classifiers have as few as three support vectors and more than half of them have zero test loss. All of this suggests the existence of a small subset of words which can distinguish among most of these models.

The predicted models Kumar [13], Middendorf-Ziv (MZ) [14], and Sole [15] are based on very similar mechanisms of iterated duplication and mutation. The model by Kumar *et al*. was originally meant to explain various properties of the WWW. It is based on a duplication mechanism, where at every iteration a prototype for the newly introduced node is chosen at random, and connected to the prototype's neighbors or other randomly chosen nodes with probability *p*. It is therefore built on an imperfect copying mechanism which can also be interpreted as duplication-mutation, often evoked when considering genetic and protein-interaction networks. Sole is based on a similar idea, but is an undirected model, and allows for two free parameters, a probability controlling the number of edges copied and a probability controlling the number of random edges created. MZ is essentially a directed version of Sole. Moreover, we observe that none of the biological networks were predicted to be generated by preferential attachment even though these networks exhibit power-law degree distributions. The duplication-mutation schemes arise as the most successful. However, it is interesting to note that every duplication-mutation model by construction gives rise to an *effective *preferential attachment [16]. Our classification results therefore do not dismiss the idea of preferential attachment, but merely the specific model which directly implements this idea.

**Figure 1:**
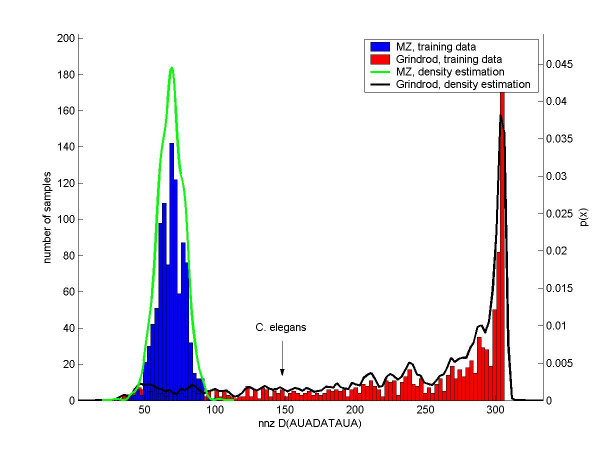
***C. elegans*: kernel density estimation for the word nnz ***D*(*AU AD AT AU A*). Data for two different models are shown: the Middendorf-Ziv [14] model and the Grindrod [17] model. *C. elegans *is robustly classified as a Middendorf-Ziv network. The Grindrod model is the runner-up. We here show data for a word that especially *disfavors *the Middendorf-Ziv model over the Grindrod model. The histograms of the word over the training data are shown along with their associated densities calculated from the data by Gaussian kernel density estimation. The densities give the following log-*p*-values at the word value for the *C. elegans *network: log(*p*_*MZ*_) = -376, log(*p*_*Grindrod*_) = -6.23.

**Figure 2:**
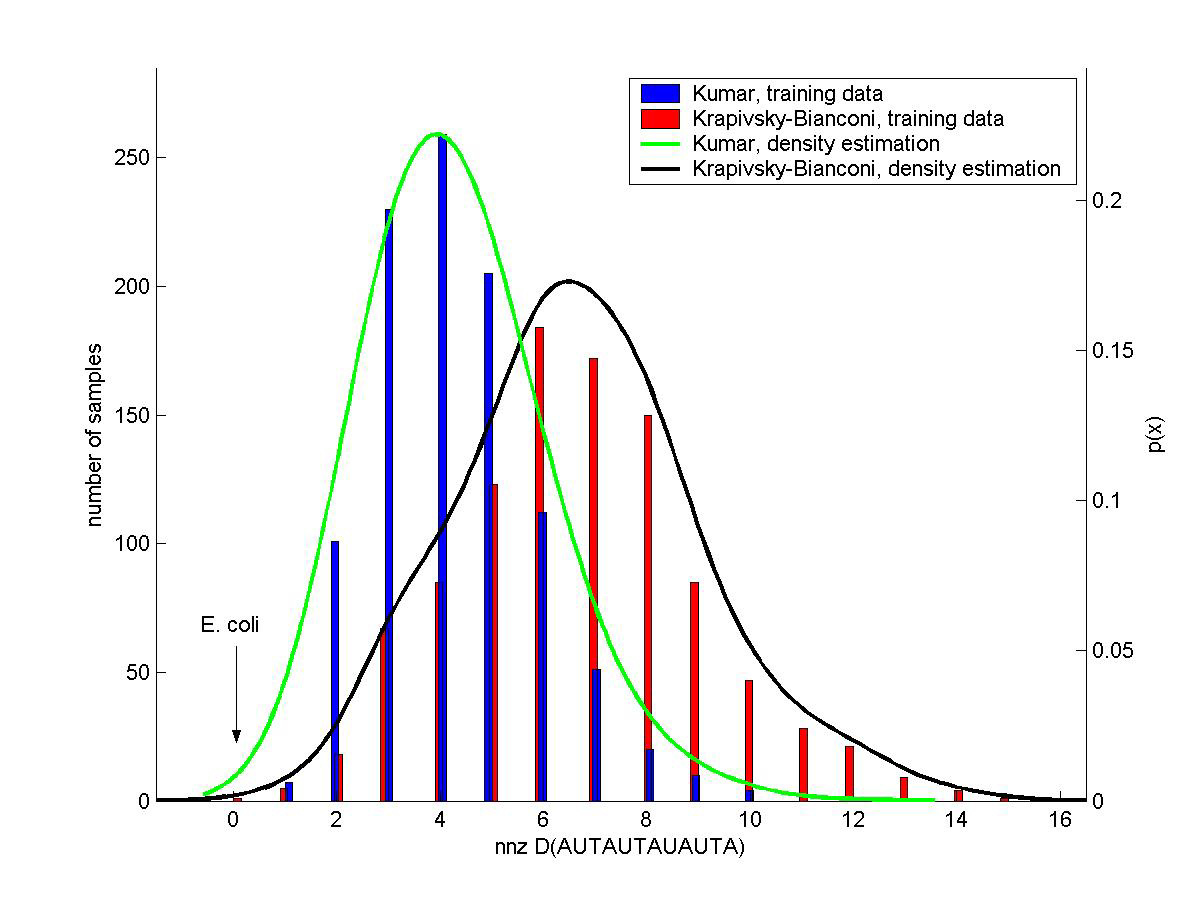
***E. coli*: kernel density estimation for the word nnz ***D*(*AUT AUT AU AUT A*). Data for two different models are shown: the Kumar model [13] and the Krapivsky-Bianconi [18, 14] model. *E. coli *is robustly classified as a Kumar network. The Krapivsky-Bianconi model is the runner-up. We here show data for a word that especially *favors *the Kumar model over the Krapivsky-Bianconi model. The histograms of the word over the training data are shown along with their associated densities calculated from the data by Gaussian kernel density estimation. The densities give the following log-*p*-values at the word value for the *E. coli *network: log(*p*_*Kumar*_) = -4.22, log(*p*_*KB*_) = -12.0.

Kumar and MZ were classified with almost perfect robustness (see Subsection Robustness under Methods) against 500-dimensional (out of 4680 dimensions) subspace sampling. With 26 different choices of subspaces, *E. coli *was always classified as Kumar. We therefore assess with high confidence that Kumar and MZ come closest to modeling *E. coli *and *C. elegans*, respectively. In the case of Sole and the *S. cerevisiae *protein network we observed fluctuations in the assignment to the best model. 3 out of 22 times *S. cerevisiae *was classified as Vazquez (duplication-mutation), other times as Barabasi (preferential attachment), Klemm (duplication-mutation), Kim (scale-free static), or Flammini (duplication-mutation) depending on the subset of words chosen. This clearly indicates that different features support different models. Therefore the confidence in classifying *S. cerevisiae *to be Sole is limited. The statistical significance of individual words in different models is investigated using kernel density estimation (see Methods) by finding words which maximize *η*_*ij *_≡ *p*_*i*_(*x*_0_)/*p*_*j*_(*x*_0_) for two different models (*i *and *j*) at a word value of the real data set *x*_0_. Figure 1 shows training data for two different models used to classify the *C. elegans *network: the MZ model [14] which wins in the classification results, and the runner-up Grindrod model [17]. The histograms for the word nnz *D*(*AU AD AT AU A*) are shown along with their estimated densities, nnz *D*(*AU AD AT AU A*) extremely disfavors the winning model over its runner-up (minimizes *η*_*ij*_). The opposite case is shown in Figure 2 for *E. coli*, where the plotted word distribution supports the winning model (Kumar [13]) and disfavors (maximizes *η*_*ij*_) the runner-up Krapivsky-Bianconi model [18,14] (preferential attachment). More specifically we are able to verify that the likelihood to generate a network with *E. coli*'s word values is highest for the Kumar model for most of the words. Indeed, out of 1897 words taking at least 2 integer values for all of the models, the estimated density at the *E. coli *word value was highest for Kumar in 1297 cases, for Krapivsky-Bianconi [18,14] in 535 cases and for Krapivsky [18] in only 65 cases.

**Figure 3:**
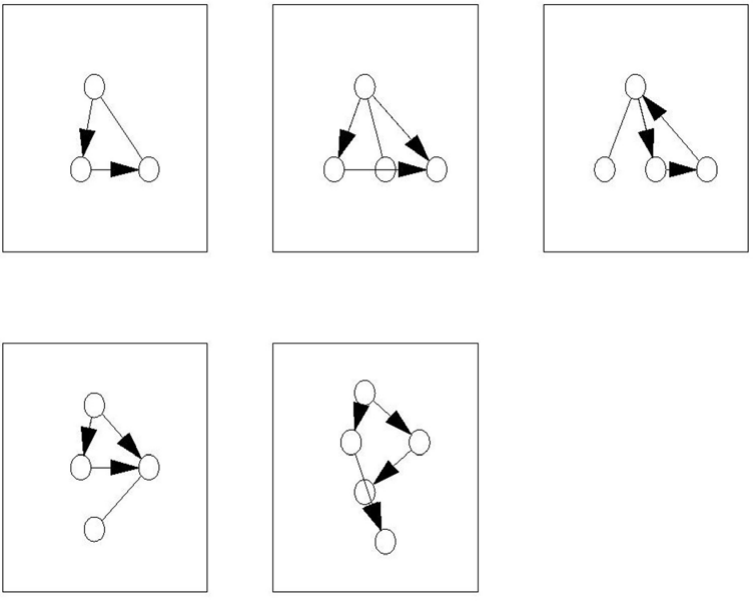
**Subgraphs associated with nnz ***D AUT AUT AU AUT A*. Every word can be associated with a set of subgraphs. If the word has a non-zero value for a given network, at least one of these subgraphs must appear. The figure shows the subgraphs associated with the word nnz *D AUT AUT AU AUT A*.

Figure 2 shows the distributions for the word nnz *D*(*AUT AUT AU AUT A*) which had a maximum ratio of probability density of Kumar over that of Krapivsky-Bianconi at *E. coli*'s word value. In fact, *E. coli *has a zero word count, meaning that none of the associated subgraphs shown in Figure 3 actually occur in *E. coli*. Four of those subgraphs have a mutual edge which is absent in the *E. coli *network and also impossible to generate in a Kumar graph. Krapivsky-Bianconi graphs allow for mutual edges which could be one of the reasons for a higher count in this word. Another source might be that the fifth subgraph showing a higher order feed-forward loop is more probable to be generated in a Krapivsky-Bianconi graph than in a Kumar graph. This subgraph also has to be absent in the *E. coli *network since it gives a zero word value, demonstrating that both the Kumar and Krapivsky-Bianconi models have a tendency to give rise to a topological structure that does not exist in *E. coli*. This analysis gives an example of how these findings are useful in refining network models and in deepening our understanding of real networks. For further discussions refer to our website. [14]

**Figure 4:**
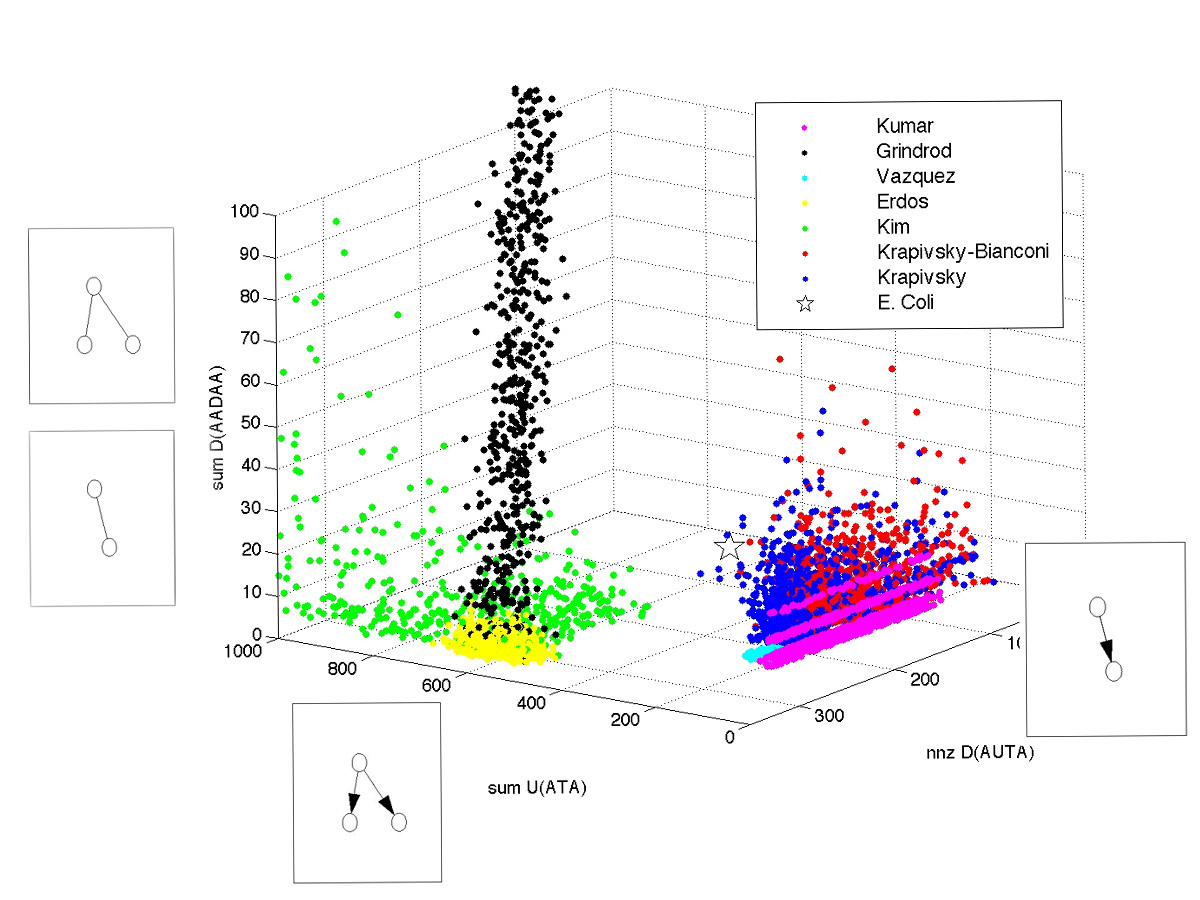
**Distributions of the *E. coli *training data in word space. **The training data for *E. coli *for seven directed models is visualized in a 3-dimensional subspace of word space. The three chosen words were found to be most discriminative according to a word ranking method. Every color is associated with a different model. The point which is occupied by *E. coli *is also indicated. The axis correspond to words which can be associated with sets of subgraphs. If a network has a non-zero word value it must possess at least one of these subgraphs.

**Figure 5:**
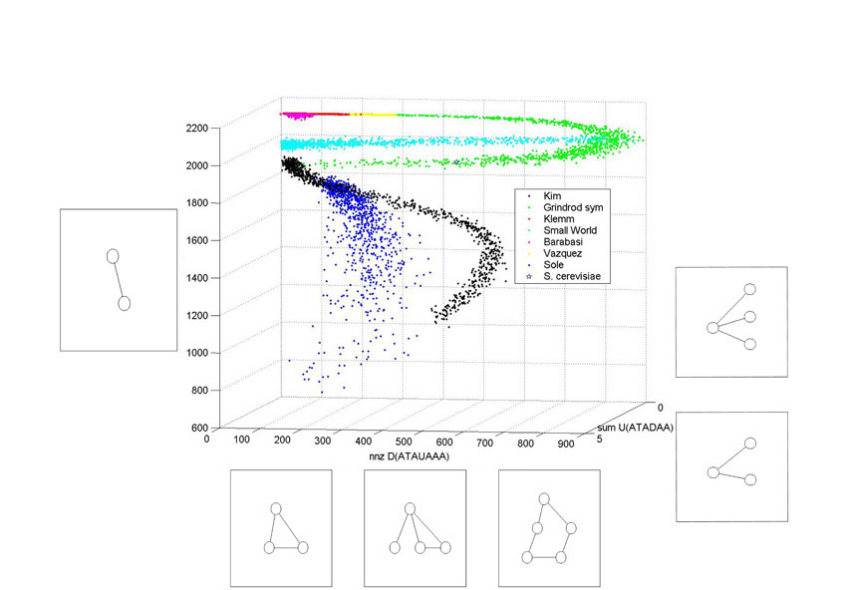
**Distributions of the *S. cerevisiae *training data in word space. **The training data for *S. cerevisiae *for seven undirected models is visualized in a 3-dimensional subspace of word space. The three chosen words were found to be most discriminative according to a word ranking method. Every color is associated with a different model. The point which is occupied by *S. cerevisiae *is also indicated. The axis correspond to words which can be associated with sets of subgraphs. If a network has a non-zero word value it must possess at least one of these subgraphs.

The SVM results suggest that one may only need a small subset of words to separate most of the models. The simplest approach to find such a subset is to look at every word for a given pair of models and compute the best split, then rank words by lowest loss. We find that among the most discriminative words some occur very often, such as, nnz (*AA*) or nnz (*AT A*), which count the pairs of edges attached to the same vertex and either pointing in the same direction or pointing away from each other, respectively. Other frequent words include nnz *D*(*AA*), nnz *D*(*AT A*) and Σ*U*(*AT A*). Figures 4 and 5 show scatter-plots of the training data using the most discriminative three words.

## Conclusions

We proposed a method to discriminate different network topologies, and we showed how to us the resulting classifier to assess which model out of a set of given network models best describes a network of interest. Moreover, the systematic enumeration of countably infinite features of graphs can be successfully used to find new metrics which are highly efficient in separating various kinds of models. Our method is a first step towards systematizing network models and assessing their predictability, and we anticipate its usefulness for a number of communities.

## Methods

### Network models

We sample training data for undirected graphs from six growth models, one scale-free static model [19-21], a small-world model [22], and the Erdös-Rényi model [23]. Among the six growth models two are based on preferential attachment [24,25], three on a duplication-mutation mechanism [16,15], and one on purely random growth [26]. For directed graphs we similarly train on two preferential attachment models [18], two static models [17,27,20], three duplication-mutation models [13,28], and the directed Erdös-Rényi model [23]. More detailed descriptions and source code are available on our website [14].

For the (directed) *E. coli *transcriptional network and the (directed) *C. elegans *neuronal network we sample training data for all directed models; for the (undirected) *S. cerevisiae *protein interaction network we sample data for all undirected models. The set of undirected models includes two symmetrized versions of originally directed models [17,28]. One should note that properties of a directed model can differ significantly from its symmetrized version. In general, the more network classes allowed, the more completetely word space is explored, and therefore the more specific the classification can be.

In order to classify real data, we sample training examples of the given models with a fixed total number of nodes *N*_0_, and allow a small interval *I*_*M *_of 1–2% around the total number of edges *M*_0 _of the considered real data set. All additional model parameters are sampled uniformly over a given range (which is specified by the model's authors in most cases, and can otherwise be given reasonable bounds). Such a generated graph is accepted if the number of edges *M *falls within the specified interval *I*_*M *_around *M*_0_, thereby creating a distribution of graphs associated with each model which should best describe the real data set with given *N*_0 _and *M*_0_.

Some of the models can be described as a generalization of another model. Although a generalized model can overlap with a specific one in its support, word space is sufficiently high-dimensional that such confusing realizations are practically impossible. To build intuition, consider that the Erdös model itself includes all possible network topologies. Nonetheless there is extremely low test loss with any other models, indicating that it still defines a particular volume in this high-dimensional space. Similarly, very few real networks have non-negligible prediction scores for being classified as Erdös networks.

### Words

The input space used for classifying graphs was introduced in our earlier work [6] as a technique for finding statistically significant features and subgraphs in naturally occurring biological and technological networks. Given the adjacency matrix *A *representing a graph (*i.e*., *A*_*ij *_= 1 iff there exists an edge from *j *to *i*), multiplications of the matrix count the number of walks from one node to another (*i.e*., [*A*^*n*^]_*ij *_is the number of unique walks from *j *to *i *in *n *steps). Note that the adjacency matrix of an undirected graph is symmetric. The topological structure of a network is characterized by the number of open and closed walks of given length. Those can be found by calculating the diagonal or non-diagonal components of the matrix, respectively. For this we define the projection operation *D *such that

**Figure 6:**
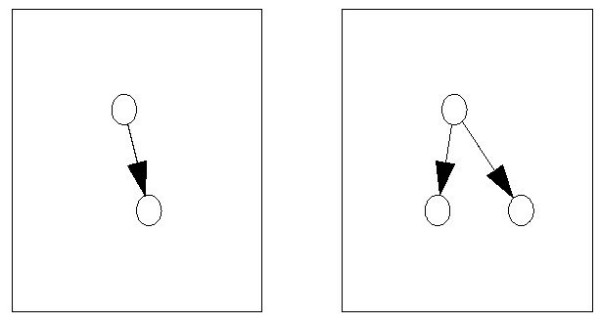
**Example for a word and its associated subgraphs. **Every word can be associated with a set of subgraphs. If the word has a non-zero value for a given network, at least one of these subgraphs must appear. The figure shows the subgraphs associated with the word nnz *AT A*. The elements of the matrix *AT A *count these two walks. *T A *corresponds to one step "up" the graph, the following *A *to one step "down". The last node could be either the same as the starting node as in the first subgraph (accounted for by the diagonal part *D AT A*) or a different node as in the second subgraph (accounted for by the non-diagonal part *U AT A*).

[*D*(*A*)]_*ij *_= *A*_*ij*_*δ*_*ij *_    (1)

and its complement *U *= *I *- *D*. (Note that we do *not *use Einstein's summation convention. Indices *i *and *j *are not summed over.) We define the primitive alphabet {*A*; *T*, *U*, *D*} as the adjacency matrix *A *and the operations *T*, *U*, *D *with the transpose operation *T*(*M*) ≡ *M*^*T*^, for any matrix *M *. *T*(*A*) and *A *distinguish walks "up" the graph from walks "down" the graph. From the *letters *of this alphabet we can construct *words *(a series of operations) of arbitrary length. A number of redundancies and trivial cases can be eliminated (for example, the projection operations satisfy *DU *= *UD *= 0) leading to the operational alphabet {*A*, *AT*, *AU*, *AD*, *AUT*}. The resulting word is a matrix representing a set of possible walks, which can be enumerated. An example is shown in Figure 6.

Each word determines two relevant statistics of the network: the number of distinct walks and the number of distinct pairs of endpoints. These two statistics are determined by either summing the entries of the matrix (sum) or counting the number of nonzero elements (nnz) of the matrix, respectively. Thus the two operations sum and nnz map words to integers. This allows us to plot any graph in a high-dimensional data space: the coordinates are the integers resulting from these path-based functionals of the graph's adjacency matrix.

The coordinates of the infinite-dimensional data space are given by integer-valued functionals

*F*(*L*_1_*L*_2_...*L*_*n*_*A*)     (2)

where each *L*_*i *_is a letter of the operational alphabet and *F *is an operator from the set {sum, sum*D*, sum*U*, nnz, nnz *D*, nnz *U*}. We found it necessary only to evaluate words with *n *≤ 4 (counting all walks up to length 5) to construct low test-loss classifiers. Therefore, our word space is a 6 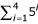 = 4680-dimensional vector space, but since the words are not linearly independent (*e.g*., sum*U *+ sum*D *= sum), the dimensionality of the manifold explored is actually much smaller. However, we continue to use the full data space since a particular word, though it may be expressed as a linear combination of other words, may be a better discriminator than any of its summands.

In [6], we discuss several possible interpretations of words, motivated by algorithms for finding subgraphs. Previously studied metrics can sometimes be interpreted in the context of words. For example, the *transitivity *of a network can be defined as 3 times the number of 3-cycles divided by the number of pairs of edges that are incident on a common vertex. For a loopless graph (without self-interactions), this can also be calculated as a simple expression in word space: sum(*D A A A*)/sum(*U AA*). Note that this expression of transitivity as the quotient of two words implies separation in two dimensions rather than in one. However, there are limitations to word space. For example, a similar measure, the *clustering coefficient*, defined as the average over all vertices of the number of 3-cycles containing the vertex divided by the number of paths of length two centered at that vertex, cannot be easily expressed in word space because vertices must be considered individually to compute this quantity. Of course, the utility of word space is not that it encompasses previously studied metrics, but that it can elucidate new metrics in an unbiased, systematic way.

### SVMs

A standard classification algorithm which has been used with great success in myriad fields is the *support vector machine*, or SVM [29]. This technique constructs a hyperplane in a high-dimensional feature space separating two classes from each other. Linear kernels are used for the analysis presented here; extensions to appropriate nonlinear kernels are possible.

We rely on a freely available C-implementation of SVM-Light [30], which uses a working set selection method to solve the convex programming problem with Lagrangian


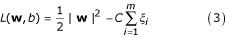


with *y*_*i*_(**w**·**x**_*i *_+ *b*) ≥ 1 - *ξ*_*i*_; *i *= 1,..., *m *where *f*(**x**) = **w**·**x **+ *b *is the equation of the hyperplane, **x**_*i *_are training examples and *y*_*i *_∈ {-1, +1} their class labels. Here, *C *is a fixed parameter determining the trade-off between small errors *ξ*_*i *_and a large margin 2/|**w**|. We set *C *to a default value 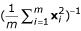. We observe that training and test losses have a negligible dependence on *C *since most test losses are near or equal to zero even in low-dimensional projections of the data space.

### Robustness

Our objective is to determine which of a set of proposed models most accurately describes a given real data set. After constructing a classifier enjoying low test loss, we classify our given real data set to find a 'best' model. However, the real network may lie outside of any of the sampled distributions of the proposed models in word space. In this case we interpret our classification as a prediction of the least erroneous model.

We distinguish between the two cases by noting the following: Consider building a classifier for apples and oranges which is then faced with a grapefruit. The classifier may then decide that, based on the feature size the grapefruit is an apple. However, based on the feature taste the grapefruit is classified as an orange. That is, if we train our classifier on different subsets of words and always get the same prediction, the given real network must come closest to the predicted class based on any given choice of features we might look at. We therefore define a *robust classifier *as one which consistently classifies a test datum in the same class, irrespective of the subset of features chosen. And we measure *robustness *as the ratio of the number of consistent predictions over the total number of subspace-classifications. In this paper we consider robustness for a subspace dimensionality of 500, a significantly small fraction of the total number of dimensions 4680.

### Kernel density estimation

A generative model, in which one estimates the distribution from which observations are drawn, allows a quantitative measure of model assignment: the probability of observing a given word-value given the model. For a robust classifier, in which assignment is not sensitively dependent on the set of features chosen, the conditional probabilities should consistently be greatest for one class.

To identify significant features we perform density estimations with Gaussian kernels for each individual word, allowing calculation of *p*(*C *= *c*|*X*_*j *_= *x*), the probability of being assigned to class *c *given a particular value *x *of word *j*. By comparing ratios of likelihood values among the different models, it is therefore possible, for the case of non-robust classifiers, to determine which of the features of a grapefruit come closest to an apple and which features come closest to an orange.

We compute the estimated density at a word value *x*_0 _from the training data *x*_*i *_(*i *= 1,..., *m*) as


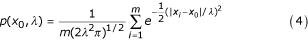


where we optimize the smoothing parameter *λ *by maximizing the average log-probability *Q *of a hold-out set using 5-fold cross-validation. More precisely, we partition the training examples into 5-folds 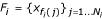, where {*f*_*i*_(*j*)}_*j *_is the set of indices associated with fold *i *(*i *= 1...5). We then maximize


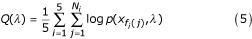


as a function of *λ*. In all cases we found that *Q*(*λ*) had a well pronounced maximum as long as the data was not oversampled. Because words can only take integer values, too many training examples can lead to the situation that the data take exactly the same values with or without the hold-out set. In this case, maximizing *Q*(*λ*) corresponds to *p*(*x*, *λ*) having single peaks around the integer values, so that *λ *tends to zero. Therefore, we restrict the number of training examples to 4*N*_*v*_, where *N*_*v *_is the number of unique integer values taken by the training set. With this restriction *Q*(*λ*) showed a well-pronounced maximum at a non-zero *λ *for all words and models.

### Word ranking

The simplest scheme to find new metrics which can distinguish among given models is to take a large number of training examples for a pair of network models and find the optimal split between both classes for every word separately. We then test every one-dimensional classifier on a hold-out set and rank words by lowest test loss.

### Web supplement

Additional figures, more detailed description of the network models, and detailed results can be found at .

### Source code

Source code was written in MATLAB and is downloadable from our our website .

## Authors' contributions

MM, EZ, and CW had the original ideas for this paper. CW and LC guided the project. Most of the coding was done by MM and EZ. CA, JH, RK, CL, and GW coded most of the network generation agorithms. The final manuscript was mainly written by MM, EZ, CW, and LC.

## Figures and Tables

**Table 1:** Summary of classification results. Results of multi-class SVM: the empirical training loss <*L*_*tr*_> averaged over all pairwise classifiers, the average empirical test loss <*L*_*tst*_>, the average number of support vectors <*N*_*sv*_>, and the winning model (with the highest number of votes from all pairwise classifiers). For the definition of robustness see Methods.

	E. coli	C. elegans	S. cerevisiae
*L*_*tr*_	1.6%	0.5%	2.1%
*L*_*tst*_	1.6%	0.5%	1.8%
*N*_*sv*_	109	51	106
Winner	Kumar [13]	MZ [14]	Sole [15]
Robustness	1.0	0.97	0.64
